# The moral experiences of children with osteogenesis imperfecta

**DOI:** 10.1177/09697330221105635

**Published:** 2022-07-08

**Authors:** Yi Wen Wang, Franco A Carnevale, Maria Ezcurra, Khadidja Chougui, Claudette Bilodeau, Sophia Siedlikowski, Argerie Tsimicalis

**Affiliations:** 5620 McGill University, Canada; 70357 Shriners Hospital for Children®-Canada, Canada; 70357 Shriners Hospital for Children®-Canada, Canada; 5620 McGill University, Canada; 70357 Shriners Hospital for Children®-Canada, Canada

**Keywords:** Art, child ethics, agency, moral experience, qualitative research, chronic illness, disability

## Abstract

**Background:**

Serious ethical problems have been anecdotally identified in the care of children with osteogenesis imperfecta (OI), which may negatively impact their *moral experiences*, defined as their sense of fulfillment towards personal values and beliefs.

**Research aims:**

To explore children’s actual and desired participation in discussions, decisions, and actions in an OI hospital setting and their community using art-making to facilitate their self-expression.

**Research design:**

A focused ethnography was conducted using the moral experiences framework with data from key informant interviews; participant observations, semi-structured interviews, and practice-based research (art-making) with 10 children with OI; and local documents.

**Participants and research context:**

The study was conducted at a pediatric, orthopedic hospital.

**Ethical considerations:**

This study was approved by McGill University Institutional Review Board.

**Findings/results:**

Children expressed desires to participate in their care, but sometimes lacked the necessary resources and encouragement from healthcare providers. Art-making facilitated children’s voice and participation in health-related discussions.

**Conclusions:**

Healthcare providers are recommended to consider the benefits of art-making and educational resources to reduce discrepancies between children’s actual and desired participation in care and promote positive moral experiences.

## Introduction

Osteogenesis Imperfecta (OI) is a rare genetic disorder that causes connective tissue weakening and fragile bones prone to fractures, breaks, and injuries.^1–3^ It affects approximately 1 in 10,000 individuals in North America and can lead to varying degrees of physical limitations (e.g., mild to severe bone deformities, use of orthopedic braces or wheelchairs for mobility),^3^ acute and chronic pain,^4^ and quality of life concerns (e.g., fear of fracture, discrimination, and repeated hospitalizations).^3,5^ Although OI treatments can reduce bone fragility through use of bisphosphonates, orthopedic surgery, and physical/occupational therapy,^6^ there is little evidence of improvements in pain, mobility, and quality of life.^7^ Given the rarity of OI and the primary focus of scientific literature on medical/pharmacological outcomes, the lived experiences of children OI remain largely ‘unknown’ to healthcare providers (HCPs), who may lack clear OI practice guidelines, policies, and knowledge to meet these children’s needs and best interests in care. This study will help to address the aforementioned challenges by rendering the lived experiences of children with OI visible.^1,8^

## Background

Preliminary evidence suggests that children with OI confront a host of ethical concerns in their daily lives, such as unexplained injuries and unintended consequences associated with inconclusive genetic tests^9^; social isolation,^3^ feelings of “otherness,”^10^ and describing oneself as “mutants”^11^; and being frequent recipients of larger societal discourses about genetic screening,^9,12^ selective pregnancy termination,^13^ and disparaging messages related to disability.^14^ HCPs and other adults may perpetuate, exacerbate, or contribute to these ethical concerns by underestimating or disregarding children’s competencies due to their status as minors or misconceptions about disability.^1,8,10,15^ In an analysis of children’s agency, Montreuil and Carnevale^16^ found that children’s voices and agential capacities were often undervalued by healthcare measures intended to protect their best interests.

The marginalization of children in their care appears to be a common issue within diverse healthcare settings and amongst children with various health conditions, such as (but not limited to) children hospitalized for planned treatment or acute illness,^17,18^ as well as children with cancer^19,20^ or cystic fibrosis.^21,22^ For example, Coyne et al.^20^ found the input of children with cancer were rarely elicited in decisions regarding their health, leading to feelings of anger, anxiety, and frustration. Failure to involve children in their care by HCPs may be attributed to personal, environmental, and systemic influences, such as lack of knowledge regarding ways to engage with children; insufficient time or poor continuity of care, causing children’s voices to be “lost” in the process of seeking treatment; cultural expectations and beliefs (e.g., it is the clinician’s responsibility to make care recommendations and decisions); or lack of institutional policies, procedures, and resources to support clinicians in involving children in care.^19,23,24^ The United Nations Convention on the Rights of the Child states that every child has the right to self-determination, respect, non-interference, and informed decision-making.^25^ Thus, promoting children’s *moral agency* (i.e., the ability to make choices, effect change, and act in self-determining ways) in healthcare may be particularly important for children with OI, who require chronic disease management and may experience disproportionate trauma, discrimination, and mental health risks.^3,11^

Esser et al.^26^ assert that the marginalization of children in matters that affect them should be attributed to scarce opportunities for active involvement and agential expression, rather than their perceived “immaturity” by adults. Art-making, described as a *doorway* into children’s inner-worlds,^27^ can facilitate conversations about difficult experiences with vulnerable children by providing an inclusive, non-verbal avenue for self-expression and emotional-processing; bridging disparities between what children want and are able to express.^28–32^ Art-making can therefore foster the participation and agency of children with OI by allowing them to contribute to the active “shaping of their social worlds.”^26^ Given that the goal of protecting children’s best interests is to achieve the best possible outcomes for them,^16,33,34^ understanding the *moral experiences* of children with OI using art-making as a vehicle for communication may provide nurses, doctors, and other HCPs with much-needed insight regarding their lived experiences and the factors that challenge or support their moral agency, participation, and best interests.^35^ Moral experience encompasses a person’s “sense that values that [they] deem important are being realised or thwarted in everyday life”, and includes their interpretations of lived encounters that “fall on spectrums of right-wrong, good-bad or just-unjust.”^36^

## Theoretical framework

This study was guided by a moral experience framework.^36^ This framework was conceptualized from Kleinman’s conception of individual’s moral experiences and what matters to them, which are shaped by “cultural meanings, social interactions, and subjectivity (inner emotions and sense of self)”^37^; and Taylor’s conception of hermeneutics, which posits that subjective experiences, their meaning, how they matter, and what one considers moral are embedded in individuals’ unique sociohistorical–cultural background, called *horizons of significance*.^38^ This framework emphasizes children’s experiences and the context(s) they are rooted in. The “spectrums of right-wrong, good-bad or just-unjust” highlights nuances in children’s moral experiences and allows researchers to examine the experiences in moments of moral conflict/distress and seemingly mundane events.

## Aims

The aims of this study were to understand the moral experiences of children with OI using art-making to elicit their voices and explore the question: *what are children’s actual and desired participation in discussions, decisions, and actions in an OI hospital setting and community?*

## Methods

### Design and setting

Following institutional ethical approvals from McGill University, a focused ethnography was conducted at a university-affiliated, non-for-profit, pediatric orthopedic hospital in collaboration with the hospital OI program, which was founded in the 1990s and internationally acclaimed as the standard of OI care.^6^ Focused ethnographies are valuable in children’s healthcare research because it allows researchers to observe naturally-occurring events, reduce power imbalances, focus on distinct local issues, and use diverse data sources to achieve an in-depth and rich examination of children’s experiences and “the cultural meanings they use to understand these social processes.”^39–41^

### Sample and recruitment

Purposive sampling strategies were used to recruit 10 participants and 19 adult key informants (Table 1). HCPs approached families with a study information sheet. With permission, the research team explained the study to eligible participants, obtained informed consent from parent(s)/legal guardian(s), and assent from the child.

**Table 1.** Study eligibility criteria.

Criteria	Children	Key informants
Age	Ages 3–17 (inclusive)	18 years or over
Health Status	Has an OI diagnosis AND No concerns or risks to participant health as perceived by healthcare staff in relation to the study	No perceived concerns or risks to participant health in relation to the study
Involvement with OI community	Currently receiving medical follow-up at study site	Former pediatric OI patient OR Multidisciplinary healthcare team member at study site OR Parent of an OI child receiving follow-up at the study site OR Received medical care for OI at study site OR Representative of the OI community (e.g., OI foundations, community leaders)
Consent	Consent from at least one parent/guardian	Consent from participant
Language spoken	English, French, Spanish	No English, French, Spanish

OI = osteogenesis imperfecta.

### Data collection

Data collection occurred in three phases: Phase I (Preparation), Phase II (Action), and Phase III (Consolidation and Mobilization of Knowledge). Phases I and II occurred from September 2017 to April 2018 and laid the foundation for Phase III. The first two phases comprise the current study and are described in detail below. Due to the difference in research scope and aims, Phase III is presented in a separate study,^42^ but summarized briefly below.

In Phase I, a doctoral-trained art-educator assisted the research team in developing art-making activities over 7 months to address the study purpose integrating diverse media (Supplemental Table S1). During Phase I, the clinicians became accustomed to having an inaugural artist-in-residence at the hospital while the artist became familiar with the study setting, population, and research procedures. The study protocol and materials were finalized in consultation with key informants convened as an Advisory Council of clinicians at the study site. The council was later reconvened for input on data analysis, interpretation, and Phase III preparations (to be reported elsewhere).

In Phase II, data were collected using practice-based research (art-making), semi-structured interviews, participant observations, and key text retrieval. Practice-based research is a methodological approach intended to advance knowledge through original investigation and includes the invention of ideas, images, performances, and artifacts that may lead to new or improved insight.^43^ Key texts consist of locally written materials that reflect the community and provide historical context and practice standards.^44^ These documents may yield additional insights about the study context, such as how children’s moral experiences and participation are understood and practiced in the hospital and, in turn, how children’s moral experiences are embedded in their unique sociohistorical–cultural background. Texts were retrieved from the hospital units, departments, and internal/external websites in consultation with the Advisory Council.

In this study, art-making activities were used to explore children’s moral experiences. Demographic information was collected from parent(s)/legal guardian(s). Children were presented with a list of art activities by the artist but could also design their own. During or after art-making, semi-structured interviews were conducted by the artist and/or researcher in the child’s preferred language (see Supplemental Materials Interview Guide). All interviews were audio-recorded with parent and child permission. Observations, field notes, and reflections were transcribed within 48 h. Children’s artworks were photographed, and children were invited to keep or donate their artwork to the art-cart, which contained the supplies used for this research study and was later donated to the hospital for art-therapy use. Donated artworks served as examples or inspirations for other children being treated at the hospital. The activities and interviews lasted 15–65 min, varying with participants’ capacity and discussion topics. The interviews were conducted privately at the hospital during the child’s hospitalization or day-treatment, with or without a family member present depending on the child’s preference.

Phase III was initiated *after* the completion of Phases I and II, which laid the foundation for interdisciplinary collaborations with HCPs. The collaboration allowed the research to mobilize the study findings by generating action steps and practice changes addressing the moral experiences of children with OI. Phase III ultimately led to the creation of a preliminary ethical framework to optimize the participation of children with OI. This work is presented in a separate publication by Wang et al.^42^

### Data analysis

Data analysis was iterative, inductive, and continuous,^45^ and conducted in three phases led by three co-investigators. The first phase included consolidation and initial open coding of all data from observations, fieldnotes, transcripts, children’s art-making and artwork, and analytical notes guided by the primary research question, *what are children’s actual and desired participation in discussions, decisions, and actions in an OI hospital setting and community?*^45^ Key texts were tabulated, categorized per topic, and analyzed to understand how children’s voices, best interests, agency, and moral experiences were implicitly and explicitly understood and practiced. The second phase involved categorizing the codes within and between sources, generating questions for subsequent data collection and ongoing consultation between the research team and key informants. The third phase included axial coding (relating categories to each other), thematic analysis (organizing categories into themes), and comparative analysis (drawing connections between themes and categories).^46^ A conceptualization of children’s involvement in care was created to present findings based on their actual and desired participation in discussions, decisions, and actions at the study site and in their community.

## Results

### Sample characteristics

Of the 11 children approached (of varying ages and OI types), 10 assented to participate with parental consent. Five children were age 10 or below, and five children were above the age of 10. The research team consulted 19 key informants in Phase I and Phase II, who assisted in reviewing interview questions, retrieving key texts, and offering insight into findings.

### Description of context

Fifty-seven key texts were analyzed to inform our contextual understanding of the study setting. The key texts (Supplemental Table S2) included: (a) educational documents, (b) forms and orientation documents, and (c) projects and research. Two themes were identified.

#### Scarce patient educational resources rarely addressing children’s healthcare experiences

Twenty-eight of the 33 educational documents focused on parental engagement in care by providing information about OI and promoting collaboration with clinicians. The documents contained advice such as, “If you do not understand something, ask the healthcare provider to explain it to you” (Document 16). One document targeted teachers and peers of children with OI. Four documents targeted the child but mainly addressed practical aspects of care (e.g., use of brace and wheelchair safety). Only one document, created by the OI Foundation, addressed children’s informational and psychosocial needs with topics related to treatments, collaborating with clinicians, and coping with OI (Document 33). Eight consent forms and three orientation documents recognized children over 14 years of age as consenting agents.

#### New projects recognizing children’s voices, learning needs, and participation in care

Thirteen documents were retrieved from hospital projects and initiatives, all of which emphasized children’s voice, learning, and participation in care*.* Six projects enabled patients to express their voice in the healthcare setting, such as *Sisom*, an interactive tool designed to help children communicate their symptoms and facilitate shared decision-making with adults (Document 47).^10^ Three in-progress projects increased learning opportunities for children with OI including a memory card game (Document 49), a storybook about coping with illness (Document 53), and an educational coloring book (Document 54). Four studies sought to find ways to improve children’s participation in care, including Document 50, which showcased that difficult-to-access OI resources can negatively impact youth during transition to adult healthcare.^47^ All projects and research were conducted with input from interprofessional HCPs, patients, and families, reflecting the hospital’s philosophy of care. The projects generated knowledge about the complex factors that impact the well-being of children with OI, guiding future research and practice.

### Value of art-making for children’s participation in their health and community

The discussions, decisions, and actions of participants during the interviews and art activities reflected their experiences in the hospital and community. Three themes were identified.

#### Use of art-making to foster self-expression and engagement

**Figure 1.**
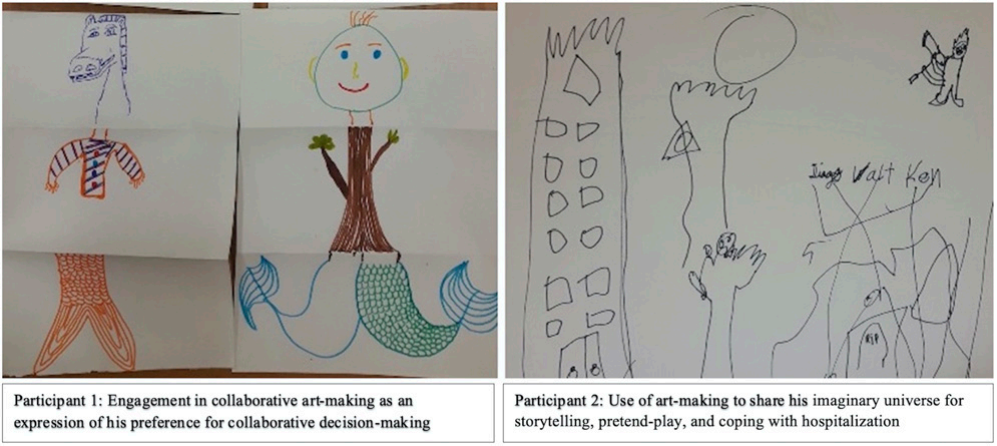
Examples of children’s art-making, which facilitated discussions about collaboration, decision-making, and storytelling. Participant 1 appeared more at ease after the researchers suggested the collaborative art activity, only then opening up about his OI experiences and family life. Conversely, Participant 2 was always eager to get back to storytelling and drawing, which was his preferred means of communication. His art-making was a doorway into his world, which was filled with imagination and OI-related metaphors, such as bones, overcoming challenges, and learning to utilize one’s “powers.”

Most participants immersed themselves in art-making by seizing the opportunity to create art, facilitating discussions about their values, beliefs, and agential expressions. Participant 2 (age 10) eagerly chose to draw an imaginary universe, which he used to share his fictional stories (Figure 1, Participant 2). Although his father was present at the beginning, he later exited the room to speak with a nurse after ensuring that Participant 2 was comfortable carrying on with the interview alone. Participant 2 appeared to be engaged and often asked researchers “can I get back to my story?”, suggesting that storytelling was an important and desired means of self-expression for him. He incorporated OI-related elements in his stories and confirmed that he gravitates towards stories in the hospital, everyday life, and school.

Participant 2: “The weirdest part is that [the characters] are all surrounded with bones. Every time they wake up, skulls, bones, whatever.”

Interviewer: “So how do you keep track of all these [stories] that you imagine? Do you write about them?”

Participant 2: “Yeah, I do! […] When I sleep at night, or when I rest, I like to think about them. My dreams.”

Interviewer: “When you’re in the hospital, do you think about these stories?”

Participant 2: “Yeah.”

Interviewer: “That’s amazing. Do you share the stories with someone?

Participant 2: “Well I was in the ten people who had the greatest stories [in school]!”

Interviewer: “What do you like most: when you have complete freedom [to write] or when the teachers [give instructions]?”

Participant 2: “Complete freedom! That’s when my imagination goes (makes a sound reminiscent of a rocket).”

Participants also sought the involvement of family and researchers, becoming more engaged when art-making was a team process. Participant 1 (age 17) was initially reluctant to discuss his OI. However, he became “interested and even relieved” (fieldnotes) when asked about engaging in a team drawing activity with the interviewers which may be attributed to the anxiety-reducing effects of art (Figure 1, Participant 1). Although Participant 1 was “mostly quiet during the exercise” (fieldnotes), he was relaxed and appeared to thoroughly enjoy the process of collaborative art-making. After the completion of the activity, he stated that he preferred the collaborative activity over individual art-making and through it, learned that “I have to start to trust more in myself and to like what I do, no matter that I’m good or bad.” His inclination towards the team drawing activity sparked discussions about his preference for collaborative decision-making.

Participant 1: “My parents’ opinions are the most important because they want the best for me. Most of my decisions I take myself and I ask others for their opinions to see how good it is.”

Interviewer: “When there is a decision to make, do you prefer teamwork?”

Participant 1: “Yes, because there are multiple opinions to help make the right choice […] I tell [my decision] to my friends, my parents, someone else so that I can get their opinion on my decision”

#### Desire and capacity for participation in health

**Figure 2.**
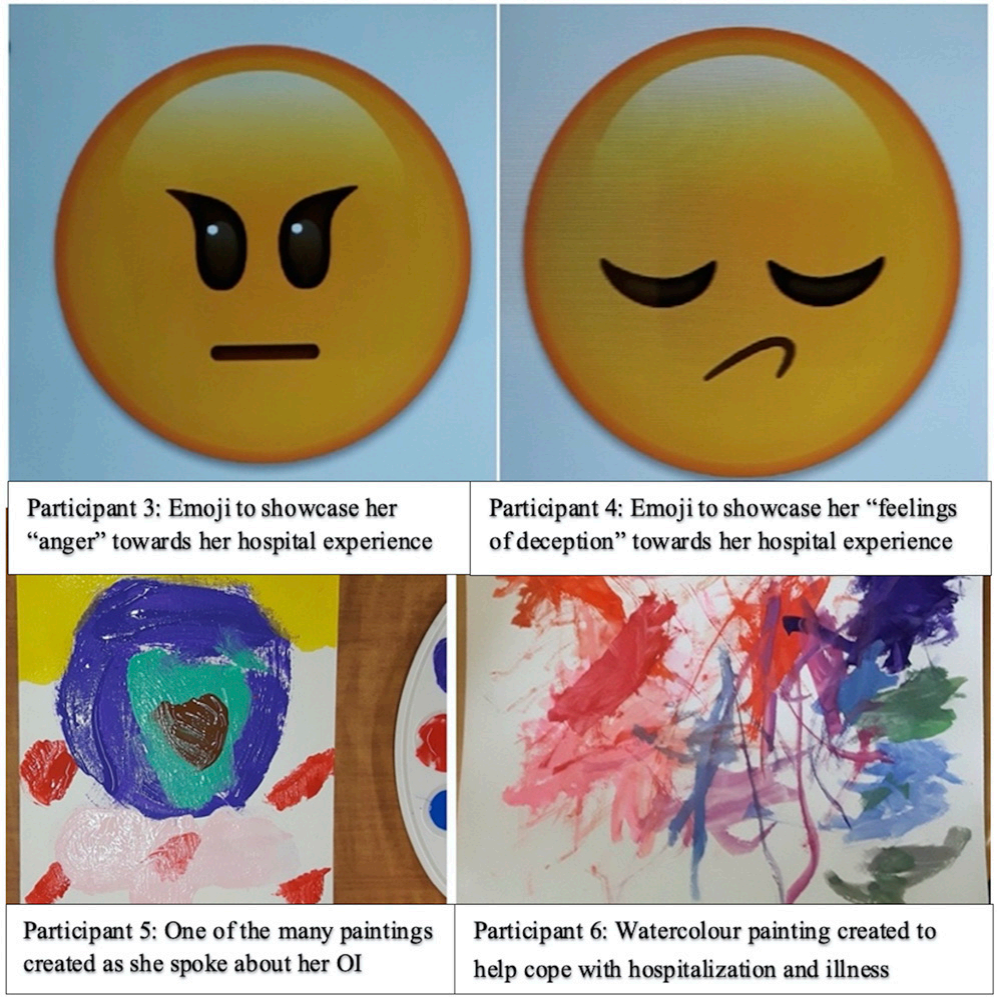
Examples of children’s art-making which prompted discussions about their OI knowledge, and their actual and desired participation in care. Participant 3 and 4’s emojis reflected their feelings of marginalization. The process of art-making helped them to process and disclose their unpleasant healthcare experiences. Participant 5 spoke extensively about her self-care capacities while using art-making as a form of play. Participant 6 avoided questions about her OI, opting to talk about her art instead. However, the conversations surrounding her art indirectly led to the disclosure of her OI knowledge and awareness.

Although most participants reported positive life experiences, they also described situations where they were excluded from health-related discussions, decisions, and actions, leading to feelings of frustration, anger, and fear. These emotions were identified by Participants 3 and 4 (both age 13) during an art-making activity (Figure 2, Participant 3 and 4), which appeared to help them become more “comfortable discussing delicate topics” (fieldnotes). Initially, both participants declined the art activities, opting solely to participate in the interviews; however, they later opted to make Emojis to help communicate and reflect on their emotions as they delved into discussions about their healthcare experiences. Their first responses were “short and vague, but changed to a fluid and rich conversation” (fieldnotes) with art-making.

Participant 3: “I didn’t expect [the surgery]—it felt imposed” and “I just had a fracture, and the discharge was rushed […] I couldn’t climb the stairs because there was snow and [the HCPs] told me to climb using my bum, but that’s not realistic.”

Participant 4: “I woke up one morning and [the doctors] told me ‘off you go, time to leave’ […] I did not really feel like I was ready to leave […] I felt pushed around […] they told me ‘don’t worry, everything will be fine’, but I felt a mix between feelings of deception, but that’s how we feel when we’re not heard.”

When asked if they had discussed their feelings regarding the medical decisions made on their behalf with a parent or clinician, Participant 3 responded, “No, because it wouldn’t have changed anything”; yet, she reported feeling “angry”, “frustrated”, and “pushed around” when describing her hospital experiences. Similarly, Participant 5 recounted feelings of distress and pain during her admission to another hospital where HCPs dismissed her voice.

Participant 5: “There were doctors that I didn’t like because they were a bit mean […] Each time I said to stop touching my leg, they didn’t listen. I didn’t like that […] I didn’t know how to manage it.”

Participant 2 reported feeling disappointed when he had to unexpectedly cancel playdates to accommodate healthcare appointments. In these collective cases, participants described not knowing how to handle these situations where they felt excluded from decisions, discussions, and actions concerning their health, suggesting that they may have lacked the necessary knowledge, skills, or confidence to assert their voices during care.

Some participants also expressed their desired healthcare changes, which may better support their participation and well-being. Participant 1, a recent immigrant whose primary language was Spanish, discussed his challenges communicating in the French and English-speaking hospital.

Participant 1: “I’m not even speaking French right, so [English] is a bit difficult”

Interviewer: “And you think that being in a hospital where they speak French makes it harder for people to understand what you want?”

Participant 1: “I think so”

Interviewer: “If people speak Spanish is it hard for you to explain yourself?”

Participant 1: “No”

Participant 1 explained that he would feel “confident” expressing himself if clinicians understood Spanish. In terms of the physical space, Participant 10 stated that while he likes the current hospital, he prefers smaller hospitals which are more “peaceful, calm, the things you associate with a home.” Finally, Participant 4 reported having no opportunities to voice her discontentment during discharge. She stated, “I wish someone took time to speak with me.”

Despite these challenges, most participants had the capacity to participate in their care. Participant 5 (age 6) and 6 (age 3) demonstrated remarkable understanding of their OI during art-making despite their young age (Figure 2, Participant 5 and 6).

Participant 6 avoided talking about her OI and appeared to use painting as a distraction from hospital stressors. She steered conversations towards her home life, artwork, and brother, for whom she was making a painting for. Despite her avoidance of OI-related topics, her response to the interviewer’s question showcased her awareness towards OI and preventing injury by sitting down while bathing.

Interviewer: “Do you think there are moments that are better for painting than others? […] When you are having a shower, is that a good time to paint?”

Participant 6: Yep. I sit down so I don’t slip.”

Participant 5 made multiple artworks individually and in collaboration with researchers while speaking openly about her experiences in the hospital. She avoided speaking about her life at home but reported learning skills such as toileting independently to avoid conflicts between her mother and grandmother (her primary caregivers) about who will help her.

Participant 5: “When I talk about OI, they bicker […] My mom tells my grandmother to help when I need to go [to the bathroom]. My grandmother is old, so it’s certain she cannot take me […] so I found a way to walk on my own. I drag myself on my bum.”

#### Valuing the support of their community

**Figure 3.**
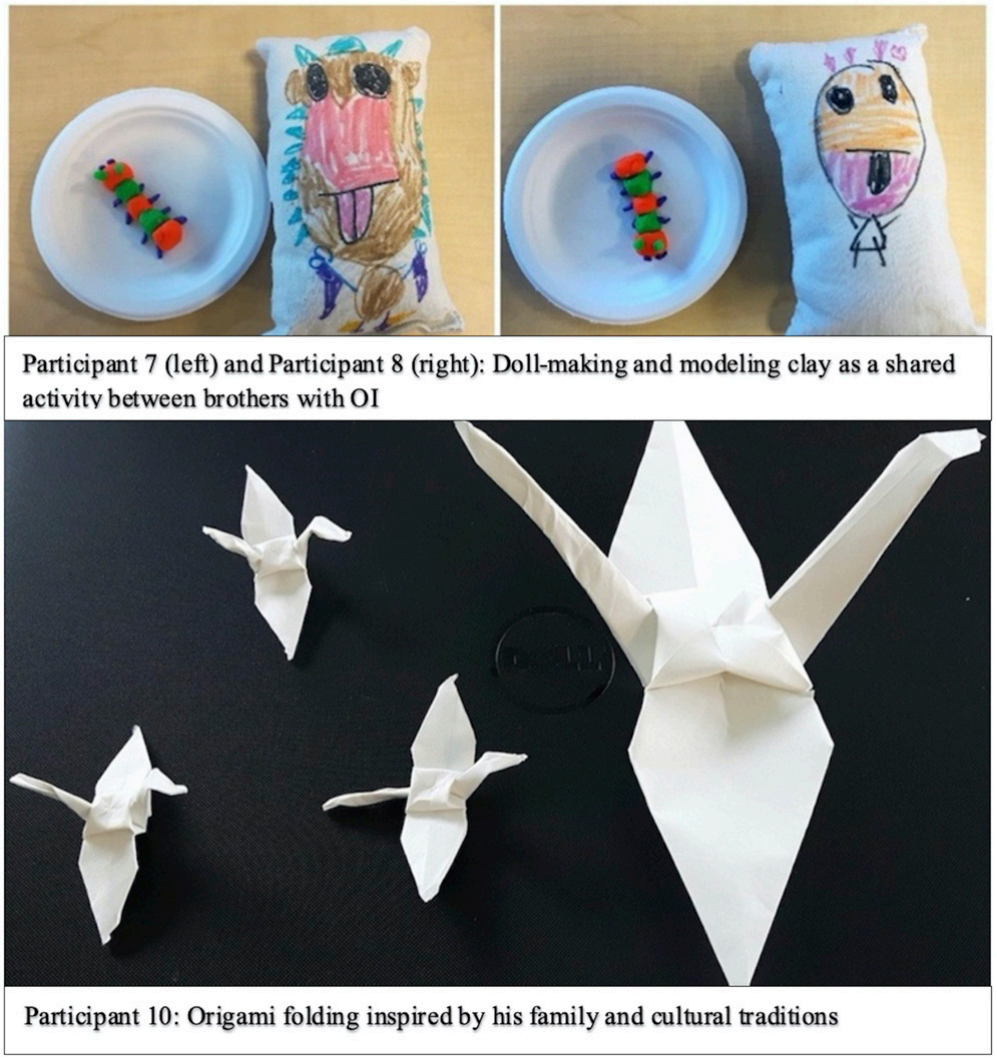
Examples of children’s art-making, which led to discussions about the importance of their families and communities. Participants 7 and 9 spoke about their parents’ support for their OI. The brothers chose similar art activities and donated their artworks to the art-cart for other children to enjoy. Participant 10 designed his own art activity by choosing to fold origami cranes, which are reflective of his culture and family traditions. The crane is a mystical creature that represents good fortune, resilience, and hope and healing during challenging times (39). In line with the positive symbolic meaning of the origami crane, Participant 10 preferred to speak about his positive experiences within his community, rather than sharing his negative OI experiences.

Most participants valued, trusted, and felt supported by their communities which consisted of family, peers, and hospital staff. Two brothers, Participant 7 (age 7) and Participant 8 (age 8), proudly explained that their father gets a tattoo each time they experience a fracture complete with the date, location, and number of the fracture, which helped him feel like “part of the gang.” They also described educating teachers and classmates about OI with help from their mother, who also has OI. Although both parents were present during the interview, the brothers engaged in art-making independently and did not require help during the process. They discussed their experiences openly and expressed knowledge of their OI with support from their mother, who helped to correct any inaccuracies. The brothers chose to do similar art activities (Figure 3, Participant 7 and 8).

Participant 7: “I had three fractures, but my brother had eight! He broke his spine.”

Mother: “Not the spine, just one vertebra.”

Participant 10 (age 13) opted to fold origami cranes, a choice that was inspired by his family and cultural traditions (Figure 3, Participant 10). His engagement in origami-folding subsequently sparked discussions about his life outside the hospital, revealing insights about his friendships, community involvement, and courage.

Participant 10: “[My friends and I] are a pretty tight group. They all know me pretty well and they’ve gotten the spiel of what OI means” and “Sled hockey is a disabled form of hockey. There’s still danger that I could fracture, but I have protective gear on”

Participant 9 (age 13) appeared “uncertain about what to do” (fieldnotes), but she engaged in health-related discussions with support from her mother and father, who encouraged her to make decisions about her artwork and helped her to answer questions about her care. This reflected how she exercises her agency in the hospital.

Interviewer: “How do you let people know when you want to do certain things in the hospital?”

Participant 9: “I just tell my parents.”

### Summary

Some participants experienced physical and emotional difficulties resulting from impediments to their desired participation in care, such as difficulties expressing themselves, scarce educational resources, and forgone opportunities by HCPs and adults to foster their engagement. Nonetheless, children expressed their agential capacities in other ways, including learning about OI, seeking support from family, and using storytelling for self-expression. During interviews where family was present, parents participated in small but meaningful ways, such as by supporting children in answering questions about their OI, offering encouragement, being present, or ensuring that their child was at ease before leaving. While participants often talked about their families, Participant 5 actively avoided this topic but reported learning self-management skills to avoid “bickering” at home, suggesting that not all families experienced or coped with children’s OI the same way. Integrating art-making into the interviews appeared to support children’s voices, leading to novel discoveries about their moral experiences in the hospital and community. Current hospital projects and research strive to enhance children’s voices, learning, and participation in care, which can be bolstered through art-making.

## Discussion

A focused ethnography was conducted to understand the moral experiences of children with OI. Art-making appeared to support children’s self-expression and engagement during interviews, highlighting children’s agential capacities, desires for participation in their healthcare, and the importance of their communities. Conversely, communication barriers and/or forgone meaningful encounters by HCPs may impede children’s agency and desired participation in care, contributing to negative moral experiences and feelings of frustration, anger, and fear. Other chronically-ill children expressed similar feelings as their involvement in care was heavily influenced by their illness severity,^48^ HCP’s perceptions of their competency or need for “protection,”^19,20^ and cultural or religious beliefs.^19^ Participation in care is crucial for children’s long-term health as persistent marginalization can impede the development of self-management skills (e.g., health knowledge, communication skills, and healthcare system navigation)^49^ and transition into adult healthcare systems,^50^ which is a common concern in children with OI.^8,51,52^ In the present study, the negativity, distress, or disengagement displayed by some participants towards their care appeared to be precipitated by the exclusion of their voice and lack of control over decisions impacting their health.

Participants reported feeling disappointed, distressed, or unheard when HCPs and other adults gave unrealistic medical advice, failed to keep them informed about their care or ignored their voices and agency, contributing to negative moral experiences and marginalization in care. Researchers found that children experienced fear and confusion when their informational needs were neglected in healthcare settings,^53,54^ but the provision of educational materials may facilitate information exchange and alleviate children’s distress.^55^ In the present study, only one educational resource addressed the diverse needs of children with OI but was distributed based on requests from families or the clinician’s discretion. The paucity of child-oriented educational resources may be reflective of the emphasis on medical and pharmacological outcomes in OI literature, as well as traditional conceptions of childhood which adhere to stage-based models of development (e.g., Piaget’s stages of cognitive development) and may paint children as less “capable” of moral reasoning and decision-making. Although stage-based models are prevalent in most professions dealing with children, they have also been criticized for (a) being based on observations of white, middle-class males or boys, leading to inadequacies when they are generalized to other genders and/or backgrounds; (b) minimizing children’s capacities by framing their differences from adults as signs of immaturity; and (c) perpetuating singular notions of what capacity, agency, and “normal” development looks like.^35,56–58^ Since OI is a chronic condition for which children are seen since birth, parents are typically considered the “most” responsible or capable in managing their child’s health; in turn, parents may be viewed as the most relevant individuals to target in relation to OI knowledge. Yet, the creation of accessible education resources for children with information on treatment, coping strategies, communication, and shared decision-making with clinicians can enhance chronically-ill children’s health knowledge, literacy, self-advocacy, and decision-making capacity, which are necessary skills to prepare them for inclusion in care and transition into adult care.^59–62^

Children’s actual and desired participation in care was also influenced by their relationship with HCPs. Zwaanswijk et al.^62^ found that children who felt comfortable with HCPs were more inclined to participate in their care. In the present study, art-making appeared to stimulate health-related discussions by distracting children from stressors and offering opportunities for familiarization with researchers. Art-making is a well-established health promotion tool commonly used to alleviate children’s pain and anxiety, support communication, and promote self-reflection and social interactions.^63–65^ Rollins et al.^66^ conducted a study in which artists and chronically-ill children engaged in interviews and collaborative art-making, allowing artists to understand children and create paintings that represented their experiences and personhood. At the unveiling, children reported feeling overjoyed, acknowledged, and appreciated. Similarly, pediatric nurses reported that incorporating play into practice led to enhanced their communication, cooperation, and ability to meet children’s need.^67^ In the present study, art-making (also a form of play) yielded similar benefits by helping children to process and express their thoughts, feelings, and experiences.

Lastly, children’s moral experiences were not defined exclusively by OI. Tsimicalis et al.^3^ identified six themes representing the psychosocial experiences of individuals with OI, including fear of fracture and isolation. Although our participants reported similar experiences, many also felt supported by their families and communities, were comfortable on their own, or overcame their fears. However, it appears that not all families experienced or coped with children’s OI the same way, as evidenced by some participants’ extensive discussions about their family lives and other participants’ reluctance to speak about the subject. This is in line with research by Dogba et al.^68^ who found multiple factors influenced how parents managed and reacted to their child’s OI, including OI severity, parents’ personal characteristics and tendencies, and the availability of social support. As individuals simultaneously influence and are influenced by their social relationships, parents’ experiences and perceptions with their child’s chronic illness can therefore further shape children’s moral experiences.^69^ Despite experiences of marginalization or lack of fulfillment in their desired participation in care, children described exercising their agency in other aspects of their lives which enabled them to realize personal values and beliefs (e.g., taking precautions in sports, playing with friends, finding ways to use the bathroom alone). Thus, the participants demonstrated resilience, courage, and the capacity to be active agents in their health and well-being. These qualities are fostered when children can build meaningful relationships with individuals “who understand OI” such as friends, family, and HCPs.^3^

### Strengths and limitations

Study strengths included the 8-month time allocated at the study site and the diverse data sources elicited for this focused ethnography. Retrieval of 57 hospital documents provided a comprehensive overview of the context in which children’s participation was embedded. Given that the “size” of focused ethnographies is ascertained by the magnitude of all elicited data, this study generated a large body of data that equaled or surpassed that of other focused ethnographies or qualitative, arts-based studies (e.g., Refs. [23,31,57]). In addition, the new cross-sector collaboration between the artist, researchers, and stakeholders led to the use of innovative theoretical and methodological approaches (which had not been used before in the setting or with the targeted population), and to the creation of art-making activities to capture children’s perspectives (available for future use; Supplemental Table S1). The methodology may also be transferable to other areas of children’s healthcare due to the study flexibility, detailed descriptions of the study context and participants’ experiences, and availability of interview guides and activities.

Study limitations included some participants’ reluctance to engage in art-making or avoidance of discussion topics, creating challenges during data collection and analysis. Nonetheless, the flexibility of the study design allowed the artist and researchers to adapt to children’s preferences. Currently, this study focused solely on the experiences of the children with OI, who constitute a sub-culture of the hospital setting and the larger OI community. At the same time, the perspectives of parents with medically-complex children are irrevocably tied to the experiences and well-being of children, siblings, and other family members.^1,15,70^ We were unable to provide detailed findings related to parents as our aims, methodologies, and funding sources were directed at eliciting children’s voices and experiences, which limited our ability to conduct a larger-scope study. However, further exploration of parents’ moral experiences using in-depth qualitative research may yield additional insights into the lives of children with OI, reveal gaps in knowledge and practice, and better prepare healthcare professionals and institutions to prioritize the well-being of families. Taking opportunities to learn about parents’ lived experiences through qualitative research is particularly important given the shortcomings of standardized measures of personal experience (e.g., quality-of-life questionnaires), which may include failure to capture individuals’ realities, lack of depth and substance, and social desirability biases leading to inaccurate results.^71,72^ These shortcomings may ultimately compromise the utility and beneficence of medical recommendations made on the basis of quantitative “research” only.^71^ Therefore, a future multi-center ethnography with children, caregivers, and healthcare professionals as well as observations, art-making activities, and time spent in different healthcare settings may yield a greater understanding of how individuals’ experiences, perspectives, and behaviors influence or are influenced by diverse social contexts, and the cultural meanings behind their actions.^41^

### Implications for practice, policy, and research

Currently, children’s participation in healthcare remains limited, thereby negatively impacting their agency and moral experiences. The study findings and arts-based methods may be integrated into practice for pediatric nurses, doctors, and other HCPs and researchers. Participants’ negative moral experiences can remind HCPs that children may express their agency in ways that do not align with adult conceptions of “competence”, but which are nonetheless ethically significant. The agential expressions of children can be rendered visible using alternative ways of “seeing” and communicating, such as art-making which can support children’s communication needs or preferences and create a safe space conducive to self-expression. For some participants (i.e., Participant 2), art-making helped to evoke sentiments which had not been vocalized before, but which may have affected HCP’s understanding of their best interests and subsequent care.

In addition, understanding children’s moral experiences can jumpstart the process of creating innovative healthcare policies, programs, and practices that promote the best interests and agency of children and families. In line with this goal, the study laid the foundation for other published and in-progress works at the hospital, including a qualitative descriptive study on the day-to-day experiences of caring for children with OI^73^; an exploration of caregivers’ views on internet-based technologies for managing their child’s OI^74^; ongoing development of a mobile health application to coordinate palliative homecare supports for family caregivers; the creation of a “found” poetry book on children’s and families’ experiences with OI^75^; and the creation of an ethical framework and clinician resource for optimizing the participation of children with OI in their healthcare, which constitutes Phase III of this three-phase focused ethnography.^42^ Future work will entail training HCPs and creating child-centered educational resources that are designed with children, HCPs, and families to tackle ethical issues in children’s healthcare; and the integration of a shared decision-making tool in the hospital setting.^10,76^ This study highlights the importance of pursuing further research concerning children’s agency, voices, and moral experiences; the impacts of integrating art-making into children’s healthcare; and the development of educational resources, policies, and programs that support the agency of children with OI and other vulnerable youths.

## Conclusion

The notion that children should participate in matters regarding their care is becoming increasingly accepted. Yet, the participation of children with OI in healthcare discussions, decisions, and actions varies greatly depending on the context, resources, and HCP practices. Children with OI often draw on courage and community support to realize their values and beliefs, but active involvement in healthcare is crucial for their well-being, moral agency, and positive moral experiences. Art-making appeared to be a useful and enjoyable way for children with OI to express their voice and optimize their participation in matters that are important to them. Given the implications of art-making for individuals, institutions, and public health, more research is needed to better understand children’s agency, best interests and moral experiences, and the impacts of art-making and education to drive change in children’s healthcare.

## Supplemental Material

Supplemental Material - The moral experiences of children with osteogenesis imperfecta Click here for additional data file. Supplemental Material for The moral experiences of children with osteogenesis imperfecta by Yi Wen Wang, Franco A Carnevale, Maria Ezcurra, Khadidja Chougui, Claudette Bilodeau, Sophia Siedlikowski and Argerie Tsimicalis in Nursing Ethics

Supplemental Material - The moral experiences of children with osteogenesis imperfecta Click here for additional data file. Supplemental Material for The moral experiences of children with osteogenesis imperfecta by Yi Wen Wang, Franco A Carnevale, Maria Ezcurra, Khadidja Chougui, Claudette Bilodeau, Sophia Siedlikowski and Argerie Tsimicalis in Nursing Ethics
