# Supplementary material for: The moral experiences of children with osteogenesis imperfecta
Source: Nurs Ethics. 2022 Jul 8;29(7-8):1773–91. doi: 10.1177/09697330221105635 (PMC9667074; doi:10.1177/09697330221105635)
Supplement: Supplemental Material - The moral experiences of children with osteogenesis imperfecta [file sj-pdf-1-nej-10.1177_09697330221105635.pdf]

Table S1

List and description of proposed art activities

Activity Name	Description
Self-Portrait	Participants are encouraged to think about what makes them unique. They can draw or paint a portrait that represents them.
Emoji Masks	Participants can make Emojis using a tablet to represent their feelings in the hospital.
Dolls and Puppets	Participants are given a small, white cushion to decorate and make into a puppet/doll. They are encouraged to describe how it reflects themselves.
The Best Part of Me	Participants can take a picture of the favorite part of themselves and paste the picture on a paper. They are asked to describe why they chose that part.
Body Maps	Using a diagram of a brain or heart, participants are encouraged to draw or write over the template to show their feelings and thoughts.
Photo Collage	Participants can take pictures of personal items they brought to the hospital, paste the pictures on a piece of paper, and draw items they wish they had.
Modeling	Participants are given modeling dough to create figurines. They may decorate their creation with materials in the art-cart.
My Favorite Place	Participants are encouraged to think about their favorite place, then to paint or draw it.
Exquisite Corpse	This is a collaborative art project: one member draws a head, then folds that section so it is concealed. The next person draws the following body part and repeats the above steps until the drawing is finished.
Sports and Games	Participants are given cardboard and other recycled materials from which to construct a game they can play while at the hospital or during the day.
Clock	Using a clock template, participants can draw or paint how they use their time during the day.
Finish that drawing	A collaborative art project where each member must draw a line continuing off of what the previous person drew.
Free Style	Using any of the materials in the art cart, participants can create anything they want.
