# Supplementary material for: The moral experiences of children with osteogenesis imperfecta
Source: Nurs Ethics. 2022 Jul 8;29(7-8):1773–91. doi: 10.1177/09697330221105635 (PMC9667074; doi:10.1177/09697330221105635)
Supplement: Supplemental Material - The moral experiences of children with osteogenesis imperfecta [file sj-pdf-2-nej-10.1177_09697330221105635.pdf]

Table S2

List of all retrieved hospital documents from study site

#	Title	Audience	Description
1.	Everyone is Special: A Story Book about Osteogenesis Imperfecta	Teachers, peers	Children's story book for learning about OI
2.	Exercise and Sports for Children with Osteogenesis Imperfecta	Caregivers	Educational text for exercise with OI
3.	Positioning, Seating and Mobility Devices for Children with Osteogenesis Imperfecta	Caregivers	Educates parents on positioning, seating, and mobility of children with OI
4.	The Daily Care of Young Children with Osteogenesis Imperfecta	Caregivers	A comprehensive booklet for daily care (e.g. feeding, clothing, family, etc.)
5.	Physiotherapy Rehabilitation for Children with Osteogenesis Imperfecta Following Femoral and Tibial Intramedullary Roddings	Caregivers	Guides parents through the surgical period
6.	Gross Motor Development of Infants with Osteogenesis Imperfecta	Caregivers	Educates caregivers on the gross motor development of infants
7.	Independence in Daily Life for Children with Osteogenesis Imperfecta	Caregivers	Promotes children's independence in activities of daily life
8.	Post-Operative Care Instructions: Inguinal Hernia Hydrocele Orchidopexy	Caregivers	Care instructions
9.	Post-Operative Care Instructions: Circumcision	Caregivers	Care instructions

Table S2 (continued)

List of all retrieved hospital documents from study site

#	Title	Audience	Description
10.	Post-Operative Care Instructions: Cast Care	Caregivers	Care instructions
11.	Post-Operative Care Instructions: Shoulder and Elbow Surgery	Caregiver	Care instructions
12.	Post-Operative Care Instructions: Arthroscopy Knee Surgery +/- Meninscectomy	Caregivers	Care instructions
13.	Instructions pour les Soins de l’Ilizarov à la Maison	Caregivers	Care instructions
14.	Instructions pour les soins d’un plâtre à la maison	Caregivers	Care instructions
15.	Information to Parents/Patients Concerning the Prevention and Nutritional Treatment of Pressure Sores	Caregivers	Prevention and treatment instructions
16.	Take an active role in your healthcare!	Caregivers	Encourages caregiver involvement in care
17.	Anterior Thorascopic Vertebral Body Tethering	Caregivers	Informative document about what it is, how it works, treatment, etc.
18.	Epiphysiodesis and Angular Correction	Caregivers	Informative document about what it is, how it works, treatment, etc.
19.	Chirurgie d’un Jour (Guide d’Information)	Caregivers	Guides caregivers through the process of surgery

Table S2 (continued)

List of all retrieved hospital documents from study site

#	Title	Audience	Description
20.	Multidisciplinary Clinic for AMC	Caregivers	Guides caregivers through clinic procedures
21.	Admission et Chirurgie le Même Jour	Caregivers	Guides caregivers through the process of same-day surgery
22.	Rehabilitation Service (Physical and Occupational Therapy)	Caregivers	Guides caregivers through service procedures
23.	Ponseti Method (Clubfoot)	Caregivers	Informative document about what it is, how it works, treatment, etc.
24.	Are X-Rays Dangerous for my Child?	Caregivers	Answers common questions
25.	The Social Worker	Caregivers	Informative document about the social worker's role in care
26.	Stratégies d'Intervention et d'Évaluation de la Douleur	Caregivers	Informative document about what it is, how it works, treatment, etc.
27.	Multidisciplinary Bedside Rounds	Caregivers	Informative document encouraging parental involvement
28.	Boston Brace for Scoliosis	Patients	Care instructions
29.	Corset Providence Pour Scoliosis	Patients	Care instructions
30.	Buckle up! For the Patient	Patients, peers	Safety considerations for patients and peers

Table S2 (continued)

List of all retrieved hospital documents from study site

#	Title	Audience	Description
31.	[WEBSITE] – Patients and Families section	Caregivers	Informative website for hospital services, procedures, preparation, etc.
32.	Growing up with OI: A Guide for Families and Caregivers	Caregivers	Comprehensive book for living with children with OI
33.	Growing up with OI: A Guide for Children	Patients	Comprehensive book for living with OI
34.	Consent to Treatment—Orthopedic Hospitals	Caregiver, child (14+)	Consent form requiring user to read information and sign
35.	Consent to Operation and Administration of Anesthetics	Caregiver, child (14+)	Consent form requiring user to write down answers to procedural questions
36.	Release of Responsibility for Temporary Leave of Absence	Parents	Consent form requiring user to read and sign
37.	Authorization to Release Information Contained in the Medical Record	Caregiver, child (14+)	Consent form requiring user to read and sign
38.	Consent for Transfusion	Caregiver, child (14+)	Consent form requiring user to read and sign
39.	Consent to Medical Photography	Caregiver, child (14+)	Consent form requiring user to read and sign
40.	Status Verification for Admissions and Out-patient	Caregiver	Asks parent/guardian for their child's health status

Table S2 (continued)

List of all retrieved hospital documents from study site

#	Title	Audience	Description
41.	Patient Transfer form	Caregiver, child (14+)	Form requiring user to provide health information and sign for transfers
42.	Exceptional Care for Exceptional Kids: Our Professional Practice Model	Clinicians	Evaluation of the FOCUSED professional practice model
43.	Fostering a Nursing Research Culture: An Integrative Review	Clinicians	Review of nursing culture in hospitals
44.	Pain Experiences of Individuals with Osteogenesis Imperfecta across the Lifespan: An Integrative Review	Clinicians, researchers, caregivers	Review of pain experiences with OI
45.	The Psychosocial Experience of Children with Osteogenesis Imperfecta: A Mixed-Method Systematic Review	Clinicians, researchers, caregivers	Determined 6 main themes for psychosocial experiences with OI
46.	The Quality of Life of Children with Osteogenesis Imperfecta and their Families	Clinicians, researchers, caregivers	Determined QoL and what affects it for OI
47.	Giving Children a Voice: Development of the Sisom App for OI	Children, Clinicians	Adaptation of the Sisom app for OI in collaboration with children with OI
48.	Exploring the Views of Osteogenesis Imperfecta Caregivers on Internet-Based Technologies	Caregivers	Exploring caregiver's usage of internet-based technologies
49.	Bones and Fractures Memory Game	Children	Learning game for children with OI

Table S2 (continued)

List of all retrieved hospital documents from study site

#	Title	Audience	Description
50.	Exploring the Self-Management Needs of Transitioning Young Adults with OI into Adult Primary Care Services	Patients	Research project exploring the needs of pediatric patients who are transitioning into adult care
51.	Development of an Evidence-Based Transfer Summary Tool for Individuals with OI Transitioning from Pediatric to Adult Primary Care	Patients	Transfer tool for patients containing health information
52.	Development of the Good2Go MyHealth Passport for Individuals with Osteogenesis Imperfecta	Patients	Transfer tool for patients containing health information
53.	The Dream Machine	Children	Storybook for children with OI
54.	OI Colour, OI Learn	Children	Colouring book for children with OI
55.	Pediatric Orthopedic Resident's Handbook	Residents	Manual for residents
56.	Clinical Orientation Manual for Nurses	New nurses	Orientation manual for nurses
57.	Clinical Ethics Decision-Making Model	Clinicians	Model for decision making
